# Association between albumin changes and prognosis in older patients with acute myocardial infarction

**DOI:** 10.3389/fmed.2024.1508868

**Published:** 2025-01-20

**Authors:** Zhi-cheng Yang, Lei Zhang, Ying-bin Xi, Gui-hua Jiang, He Lin, Hui Pan, Zhi-hao Wang

**Affiliations:** ^1^School of Nursing and Rehabilitation, Shandong University; Department of Geriatric Medicine, Qilu Hospital, Shandong University, Jinan, Shandong, China; ^2^National Key Laboratory for Innovation and Transformation of Luobing Theory; The Key Laboratory of Cardiovascular Remodeling and Function Research, Chinese Ministry of Education, Chinese National Health Commission and Chinese Academy of Medical Sciences; Department of Cardiology, Qilu Hospital of Shandong University, Jinan, Shandong, China; ^3^The Affiliated Weihai Second Municipal Hospital of Qingdao University, Weihai, Shandong, China

**Keywords:** albumin changes, acute myocardial infarction, older people, prognosis, S-shaped

## Abstract

**Background and aims:**

Acute myocardial infarction (AMI) is the leading cause of death in the world. Therefore, early identification of the prognosis of older patients with AMI are particularly urgent, and better to improve treatment. This study aimed to explore the association between albumin changes and prognosis in older patients with AMI.

**Methods:**

Outcomes included all-cause death during hospitalization, cardiac death, gastrointestinal hemorrhage, nonfatal myocardial infarction, acute heart failure, and severe arrhythmia. Multivariable-adjusted Cox regression analysis and curve fitting were used to assess the relationship between albumin changes and prognosis in patients with AMI.

**Results:**

Our study included 288 older patients with AMI. A S-shaped association between the albumin changes and mortality of patients with AMI was found. For all-cause death, we found two inflection points were − 3.27 and 0.92 g/L. On the left side of −3.27 g/L, the OR was 0.41 (OR: 0.41, 95%CI: 0.32–0.64, *p* < 0.05). On the right side of 0.92 g/L, the OR was 0.58 (OR: 0.58, 95%CI: 0.34–0.86, *p* < 0.05). The OR was 2.12 between −3.27 and 0.92 g/L (OR: 2.12, 95%CI: 1.16–6.24, *p* < 0.05). For cardiac death, two inflection points were − 3.19 and 1.17 g/L. On the left side of −3.19 g/L, the OR was 0.45 (OR: 0.45, 95%CI: 0.28–0.79, *p* < 0.05). On the right side of 1.17 g/L, the OR was 0.63 (OR: 0.63, 95%CI: 0.38–0.86, *p* < 0.05). The OR was 4.53 between −3.19 and 1.17 g/L (OR: 4.53, 95%CI: 0.90–12.52, *p* > 0.05). After adjusting for all potential covariates, albumin changes were negatively associated with gastrointestinal hemorrhage (OR: 0.87; 95%CI: 0.81–0.94, *p* < 0.001). After adjusting for all potential covariates, albumin changes were negatively associated with acute heart failure (OR: 0.86; 95%CI: 0.75–0.99, *p* = 0.046).

**Conclusion:**

Out findings showed that a S-shaped association between the albumin changes and mortality of older AMI patients, with the inflection of roughly −3.27 g/L and 0.92/L. And changes in albumin levels are negatively correlated with gastrointestinal bleeding and acute heart failure. These findings were helpful for the clinical treatment.

## 1 Introduction

Acute myocardial infarction (AMI), a type of myocardial necrosis usually caused by blockage of coronary arteries, is the leading cause of death in the world (1). Meanwhile, patients who experience AMI remain at risk of serious complications, including heart failure, arrhythmia, and other serious adverse outcomes (2, 3). Despite the advanced treatment has markedly improved the prognosis of AMI patients over the last decades, mortality in AMI patients, especially older patients, remains high (4, 5). Therefore, early identification and accurate judgment of the prognosis of older patients with AMI are particularly urgent, and this is of great significance to improve better guidance for treatment.

Albumin is a major protein in plasma and the most abundant circulating protein in the extracellular compartment. Due to its versatility and diverse array of properties, such as maintaining vascular osmotic pressure, binding and transporting varieties of endogenous or exogenous compounds, and affecting the pharmacokinetics of many drugs, it has garnered significant scientific attention over the years (6). Low serum albumin was reported to be a predictor of adverse outcomes in patients with chronic heart failure, stroke and coronary artery disease (7–9). Moreover, low serum albumin was a negative prognostic marker in AMI patients (10). However, all the existing studies on serum albumin and prognosis in AMI patients using single time points. AMI may be accompanied by complications, such as heart failure, which can expand plasma volume, impair renal and nutritional, and blunt hepatic biosynthesis. All of these will change the albumin concentration. Therefore, single measure does not account for changes in albumin level over time, thus leading to a biased estimate of the association between albumin and prognosis in patients with AMI. And changes in serum albumin concentrations over time were reported to be more powerful than single albumin determinations (11).

However, the role of the albumin changes in predicting the prognosis of older AMI patients remains unclear. Therefore, we conducted a retrospective study to address this key question. The aim of this study was to explore the correlation between albumin changes and the prognosis of older patients with AMI.

## 2 Methods

### 2.1 Data source

**Figure 1:**
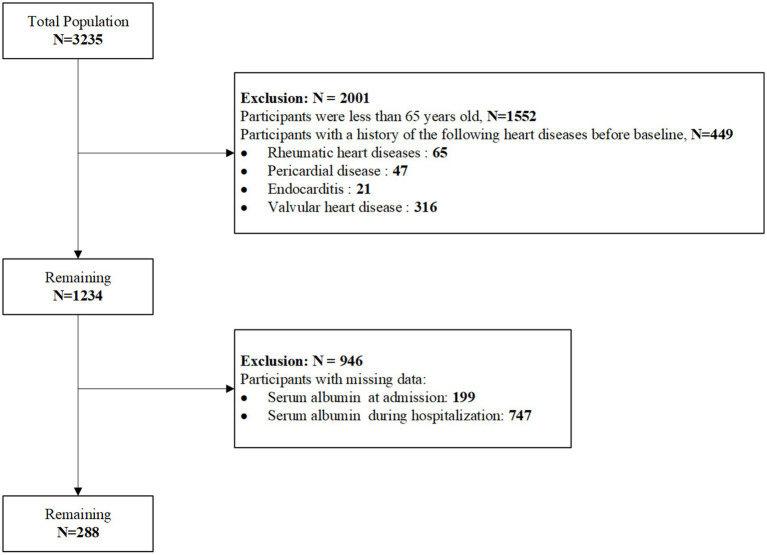
Flowchart of enrolled participants in our study.

This study is a observational retrospective study. The study included 288 patients with AMI who were hospitalized in the Department of Cardiology at Shandong University Qilu Hospital between September 2017 and March 2022. The participant selection process is shown in Figure 1. This study was approved by the Medical Ethics Committee of Shandong University Qilu Hospital (Ethics approval number: 2020092) and was conducted in accordance with the principles of the Declaration of Helsinki.

### 2.2 Inclusion and exclusion criteria

Inclusion criteria:

- Patients aged ≥65 years.
- Patients first discharge diagnosis of AMI, AMI diagnosis was defined according to the International Classification of Diseases, Tenth Revision (ICD-10) codes I21 and I22.

Exclusion criteria:

- Participants with the following cardiac conditions before baseline, including rheumatic heart disease, pericardial disease, endocarditis, valvular heart disease, myocarditis, and cardiomyopathy.
- Participants with missing albumin data.

### 2.3 Outcome

The primary outcomes were all-cause mortality and cardiac mortality.

- All-cause mortality: Defined as death due to any cause during the hospitalization period.
- Cardiac mortality: Defined as death directly attributed to cardiac causes, as documented in clinical records and death certificates. Cardiac causes include myocardial infarction, acute heart failure, fatal arrhythmias, or cardiogenic shock.

The secondary outcomes included gastrointestinal hemorrhage, nonfatal myocardial infarction, acute heart failure, and severe arrhythmias.

### 2.4 Data collection

The follow-up period began on the date of admission and ended at the earliest of the following events: death, discharge, or the conclusion of the study period (March 2022). Patient data were collected retrospectively from the hospital’s medical record management system. Basic demographic information, including age, gender, body mass index (BMI), and smoking history, was obtained. Clinical characteristics such as the type of AMI and Killip class were also recorded. Medical history and comorbidities included a history of gastrointestinal bleeding, moderate-to-severe chronic kidney disease, moderate-to-severe liver disease, hypertension, diabetes, congestive heart failure (CHF), peripheral vascular disease (PVD), and stroke. Information on medication use was documented, including aspirin, clopidogrel, ticagrelor, anticoagulants, and statins. Additional data included the length of hospital stay and whether the patient underwent percutaneous coronary intervention (PCI). Laboratory test results were also collected, including low-density lipoprotein cholesterol (LDL), serum albumin levels at admission, and albumin levels during hospitalization. The change in albumin levels during hospitalization was calculated as the difference between the average albumin level during hospitalization and the albumin level at admission. To ensure data accuracy, all information was independently reviewed and entered by two researchers. Missing data were addressed using multiple imputation to ensure the robustness of the analysis.

### 2.5 Statistical analysis

The statistical analyses in this study were conducted using SPSS version 27.0 (SPSS, Chicago, IL) and R software version 4.4.0 (R Foundation for Statistical Computing). Initially, outliers were identified and addressed using the interquartile range (IQR) method. Variables with less than 20% missing data were subjected to multiple imputation.

The study participants were categorized based on the occurrence of all-cause death during the study period and the changes in albumin levels. Continuous variables were expressed as mean ± standard deviation (SD) or median (IQR, 25th–75th percentiles) according to their distribution. Normally distributed variables were compared using independent samples t-tests, while non-normally distributed variables were compared using the Mann–Whitney U test. Categorical variables were presented as counts and percentages, and group comparisons were performed using the chi-square test. Kaplan–Meier survival curves were used to evaluate in-hospital survival rates between the two groups categorized by changes in albumin levels. The survival curves were compared using the log-rank test.

A Cox proportional hazard regression model was constructed to analyze the relationship between changes in albumin levels and outcome events. The model hypothesis was verified by martingale residual, deviation residual and schoenfeld residual analysis. The change of albumin level was fitted using restricted cubic spline curve. The change in albumin levels was set to 0 as the reference group, and the optimal model was selected based on the Akaike Information Criterion (AIC). The number of nodes was set to 4, as the AIC was the smallest at this node count. Fully adjusted models were adjusted for sex, age, BMI, smoking history, moderate-to-severe renal disease, moderate-to-severe hepatic disease, hypertension, diabetes, CHF, PVD, stroke, aspirin, clopidogrel, ticagrelor, statins, anticoagulants, ace inhibitors/angiotensin II receptor blockers (ACEI/ARB), PCI and LDL was present. Subsequently, cox or piecewise cox regression were fitted for occurrence of all-cause death, cardiac death, gastrointestinal hemorrhage, nonfatal myocardial infarction, acute heart failure, and severe arrhythmias during hospitalization to quantify associations. Subgroup analysis stratified the model by age, gender, BMI, smoking and comorbidities to observe the impact of different populations on the results. *p* < 0.05 were considered statistically significant.

## 3 Results

### 3.1 Patient characteristics

A total of 288 AMI patients who met the inclusion and exclusion criteria were enrolled in this study. The baseline characteristics of the participants are summarized in Table 1. Based on All-cause death, patients were divided into two groups: the death group (41 cases) and the survival group (247 cases). Among the total cohort, 188 (65.3%) were male and 100 (34.7%) were female, with a median age of 73 years. Patients in the death group were significantly older, had a higher proportion of Killip class ≥ II, and exhibited a higher prevalence of moderate to severe chronic kidney disease, liver disease, CHF, and stroke. Furthermore, the death group had significantly higher rates of aspirin, ACEI/ARB, and statin use, as well as a greater proportion undergoing PCI compared to the survival group. These differences were statistically significant (*p* < 0.05).

**Table 1:** Baseline characteristics of the study population according to the occurrence of all cause death.

Variables	Total (*n* = 288)	Survival group (*n* = 247)	Death group (*n* = 41)	*p*
Sex, *n* (%)				0.794
Female	100 (35)	87 (35)	13 (32)	
Male	188 (65)	160 (65)	28 (68)	
Age [Years, Median (Q1, Q3)]	73 (68, 78.25)	72 (68, 78)	75 (71, 80)	0.016
BMI [kg/m^2^, Median (Q1, Q3)]	24.22 (22.89, 27.04)	24.39 (22.89, 27.04)	24.22 (22.58, 26.87)	0.700
Smoker, *n* (%)				0.767
No	166 (58)	141 (57)	25 (61)	
Yes	122 (42)	106 (43)	16 (39)	
History of gastrointestinal bleeding, *n* (%)				0.150
No	283 (98)	244 (99)	39 (95)	
Yes	5 (2)	3 (1)	2 (5)	
AMI classification, *n* (%)				0.290
Acute Anterior MI	35 (12)	26 (11)	9 (22)	
Acute Global MI	219 (76)	190 (77)	29 (71)	
Acute Inferior MI	31 (11)	28 (11)	3 (7)	
Acute Lateral MI	2 (1)	2 (1)	0 (0)	
Acute Posterior MI	1 (0)	1 (0)	0 (0)	
Killip class, *n* (%)				< 0.001
Killip I	142 (49)	130 (53)	12 (29)	
Killip II	85 (30)	78 (32)	7 (17)	
Killip III	33 (11)	21 (9)	12 (29)	
Killip IV	28 (10)	18 (7)	10 (24)	
MSRD, *n* (%)				< 0.001
No	236 (82)	214 (87)	22 (54)	
Yes	52 (18)	33 (13)	19 (46)	
MSHD, *n* (%)				< 0.001
No	252 (88)	230 (93)	22 (54)	
Yes	36 (12)	17 (7)	19 (46)	
Hypertension, *n* (%)				0.551
No	114 (40)	100 (40)	14 (34)	
Yes	174 (60)	147 (60)	27 (66)	
Diabetes, *n* (%)				0.399
No	182 (63)	159 (64)	23 (56)	
Yes	106 (37)	88 (36)	18 (44)	
CHF, *n* (%)				0.029
No	62 (22)	59 (24)	3 (7)	
Yes	226 (78)	188 (76)	38 (93)	
PVD, *n* (%)				0.074
No	108 (38)	87 (35)	21 (51)	
Yes	180 (62)	160 (65)	20 (49)	
Stroke, *n* (%)				0.025
No	213 (74)	189 (77)	24 (59)	
Yes	75 (26)	58 (23)	17 (41)	
Aspirin, *n* (%)				0.002
No	23 (8)	14 (6)	9 (22)	
Yes	265 (92)	233 (94)	32 (78)	
Clopidogrel, *n* (%)				0.346
No	58 (20)	47 (19)	11 (27)	
Yes	230 (80)	200 (81)	30 (73)	
Ticagrelor, *n* (%)				0.118
No	222 (77)	186 (75)	36 (88)	
Yes	66 (23)	61 (25)	5 (12)	
Anticoagulants, *n* (%)				0.912
No	97 (34)	84 (34)	13 (32)	
Yes	191 (66)	163 (66)	28 (68)	
ACEI/ARB, *n* (%)				0.024
No	139 (48)	112 (45)	27 (66)	
Yes	149 (52)	135 (55)	14 (34)	
Statins, *n* (%)				0.002
No	15 (5)	8 (3)	7 (17)	
Yes	273 (95)	239 (97)	34 (83)	
PCI, *n* (%)				< 0.001
No	123 (43)	95 (38)	28 (68)	
Yes	165 (57)	152 (62)	13 (32)	
LDL [mmol/L, Median (Q1, Q3)]	2.27 (1.68, 2.96)	2.27 (1.69, 2.96)	2.05 (1.57, 2.86)	0.328
Albumin_change_level [g/L, Median (Q1, Q3)]	−0.52 (−3.21, 2)	−0.7 (−3, 2.15)	0 (−4.5, 1.35)	0.263

BMI, Body Mass Index; AMI, Acute myocardial infarction; MSRD, Moderate-to-Severe Renal Disease; MSHD, Moderate-to-Severe Hepatic Disease; CHF, Congestive Heart Failure; PVD, Peripheral Vascular Disease; ACEI, Angiotensin-Converting Enzyme Inhibitor; ARB, Angiotensin II Receptor Blocker; PCI, Percutaneous Coronary Intervention; LDL, Low Density Lipoprotein.

As shown in Supplementary Table 1, patients were categorized into the downregulated albumin level group (*n* = 164) and the upregulated albumin levels group (*n* = 124) based on changes in albumin levels. Significant differences were observed between the two groups in terms of statin use, PCI, all-cause death, cardiac death, and gastrointestinal bleeding. No significant differences were found for other variables.

### 3.2 Association between albumin changes and outcomes

**Figure 2:**
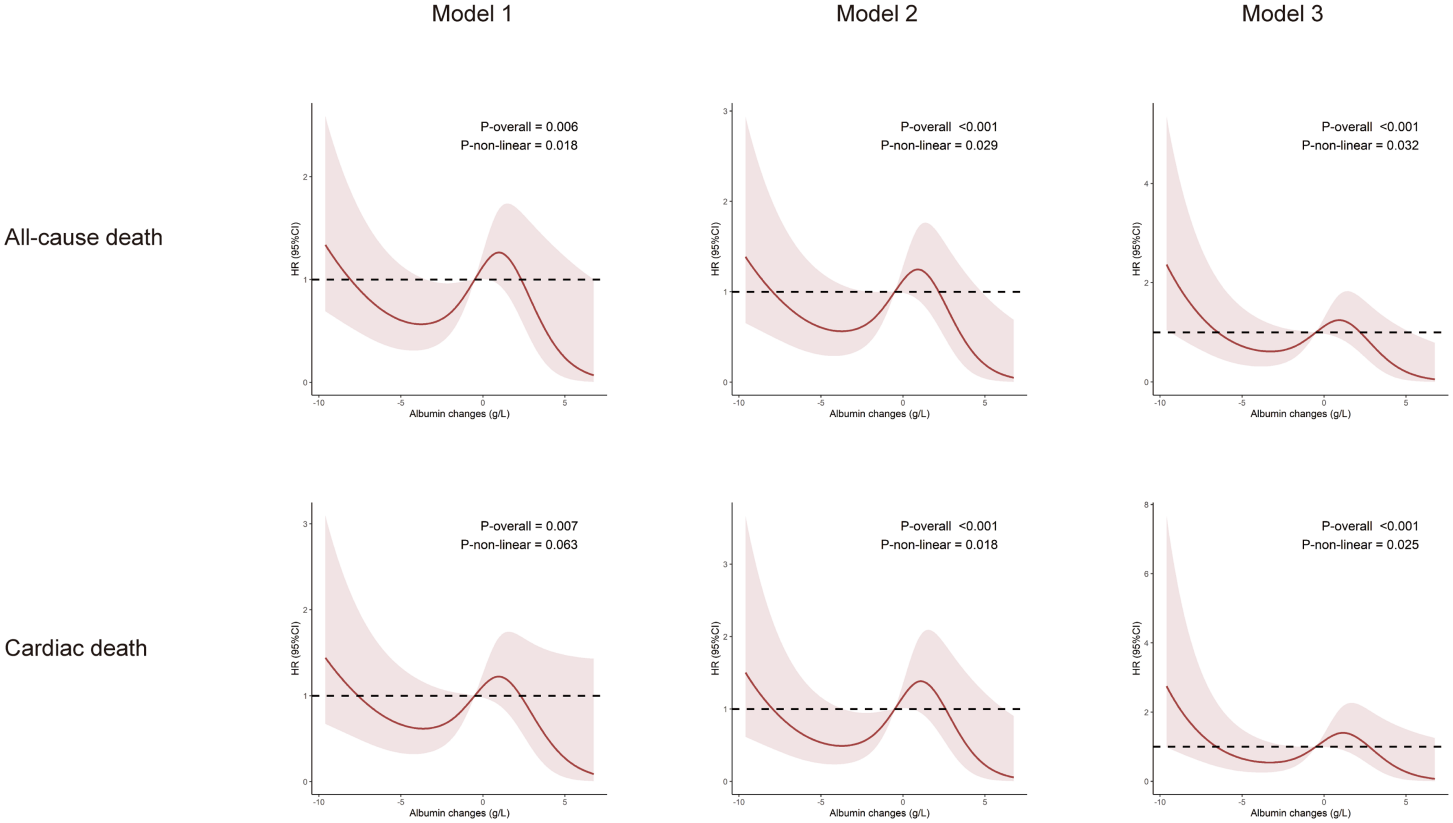
Relationship between changes in albumin levels and the incidence of all-cause death and cardiac death. Model 1: Adjusted for sex, age, BMI, and smoking history. Model 2: Adjusted for sex, age, BMI, smoking history, moderate-to-severe renal disease, moderate-to-severe hepatic disease, hypertension, diabetes, CHF, peripheral vascular disease, stroke. Model 3: Adjusted for sex, age, BMI, smoking history, moderate-to-severe renal disease, moderate-to-severe hepatic disease, hypertension, diabetes, CHF, peripheral vascular disease, stroke, aspirin, clopidogrel, ticagrelor, statins, anticoagulants, ACEI/ARB, PCI and LDL.

The follow-up period for this study ranged from 1 to 49 days (median: 11 days; interquartile range: 9–14 days). During the follow-up, the following events were recorded: 41 cases of all-cause death (14%), 32 cases of cardiac death (11%), 30 cases of gastrointestinal hemorrhage (10%), 28 cases of severe arrhythmias (10%), 12 cases of non-fatal myocardial infarction (4%), and 16 cases of acute heart failure (6%). To illustrate the outcomes of patients with different levels of albumin change, Kaplan–Meier survival curves were generated (Supplementary Figure 1). As shown in Supplementary Figure 1, the cumulative incidence of all-cause death decreased significantly with an upregulated albumin levels (log-rank test, *p* = 0.032). Through multivariate Cox regression analysis and smooth curve fitting, we found that the relationship between albumin changes and mortality of AMI patients was nonlinear (Figure 2). A S-shaped association between the albumin changes and mortality of patients with AMI was found. For all-cause death, we found two inflection points were − 3.27 and 0.92 g/L (Table 2). On the left side of −3.27 g/L, the OR was 0.41 (OR: 0.41, 95%CI: 0.32–0.64, *p* < 0.05). On the right side of 0.92 g/L, the OR was 0.58 (OR: 0.58, 95%CI: 0.34–0.86, *p* < 0.05). The OR was 2.12 between −3.27 and 0.92 g/L (OR: 2.12, 95%CI: 1.16–6.24, *p* < 0.05). For cardiac death, two inflection points were − 3.19 and 1.17 g/L. On the left side of −3.19 g/L, the OR was 0.45 (OR: 0.45, 95%CI: 0.28–0.79, *p* < 0.05). On the right side of 1.17 g/L, the OR was 0.63 (OR: 0.63, 95%CI: 0.38–0.86, *p* < 0.05). The OR was 4.53 between −3.19 and 1.17 g/L (OR: 4.53, 95%CI: 0.90–12.52, *p* > 0.05). After adjusting for all potential covariates (Table 3, fully adjusted model), albumin changes were positively associated with gastrointestinal hemorrhage (OR: 0.87; 95%CI: 0.81–0.94, *p* < 0.001; Table 3 and Supplementary Figure 1). After adjusting for all potential covariates, albumin changes were positively associated with acute heart failure (OR: 0.86; 95%CI: 0.75–0.99, *p* = 0.046; Table 3 and Supplementary Figure 1).

**Table 2:** Estimated change points in the association between albumin levels changes and Incident rate, and associations with Incident rate different change points, from Restricted Cubic Splines models.

Outcome	Albumin level change point (g/L)	Risk of outcome per 1 g/L increase in albumin below minimal change point [HR (95% CI)]	Risk of outcome per 1 g/L increase in albumin between minimal and maximum change point [HR (95% CI)]	Risk of outcome per 1 g/L increase in albumin above maximum change point [HR (95% CI)]
All-cause death	−3.27 and 0.92	0.41 (0.32–0.64)^*^	2.12 (1.16–6.24)^*^	0.58 (0.34–0.86)^*^
Cardiac death	−3.19 and 1.17	0.45 (0.28–0.79)^*^	4.53 (0.90–12.52)	0.63 (0.38–0.86)^*^

^*^p<0.05. Model adjusted for sex, age, BMI, smoking history, moderate-to-severe renal disease, moderate-to-severe hepatic disease, hypertension, diabetes, CHF, peripheral vascular disease, stroke, aspirin, clopidogrel, ticagrelor, statins, anticoagulants, ACEI/ARB, PCI and LDL.

BMI, Body Mass Index; ACEI, Angiotensin-Converting Enzyme Inhibitor; ARB, Angiotensin II Receptor Blocker; PCI, Percutaneous Coronary Intervention; LDL, Low Density Lipoprotein.

**Table 3:** Association between changes in albumin levels and risk of events during hospitalization.

Outcome	Mode1 HR (95% CI)	*p* value	Mode 2 HR (95% CI)	*p* value	Mode 3 HR (95% CI)	*p* value
Gastrointestinal hemorrhage	0.88 (0.83, 0.93)	<0.001	0.87 (0.81, 0.93)	<0.001	0.87 (0.81–0.94)	<0.001
Severe arrhythmia	0.97 (0.90, 1.04)	0.403	0.94 (0.87, 1.02)	0.143	0.92 (0.85–1.01)	0.076
Non-fatal MI	0.97 (0.84, 1.11)	0.654	0.93 (0.80, 1.09)	0.396	0.93 (0.77–1.11)	0.396
Acute Heart Failure	0.93 (0.85,1.03)	0.155	0.92 (0.81, 1.05)	0.230	0.86 (0.75–0.99)	0.046

Model 1: Adjusted for age, sex, BMI, and smoking history. Model 2: Adjusted for age, sex, BMI, smoking history, hypertension, diabetes, congestive heart failure, peripheral vascular disease, and stroke (For gastrointestinal bleeding, a history of gastrointestinal bleeding was added; For acute heart failure, corrected AMI classification and Killip classification were added). Model 3: Adjusted for age, sex, BMI, smoking history, hypertension, diabetes, congestive heart failure, peripheral vascular disease, stroke, aspirin, clopidogrel, ticagrelor, statins, anticoagulants, ACEI/ARB, and PCI (For gastrointestinal HR (95% CI) bleeding, a history of gastrointestinal bleeding was added; For acute heart failure, AMI classification and Killip classification were added).

BMI, Body Mass Index; ACEI, Angiotensin-Converting Enzyme Inhibitor; ARB, Angiotensin II Receptor Blocker; PCI, Percutaneous Coronary Intervention.

### 3.3 Subgroup analyses

**Figure 3:**
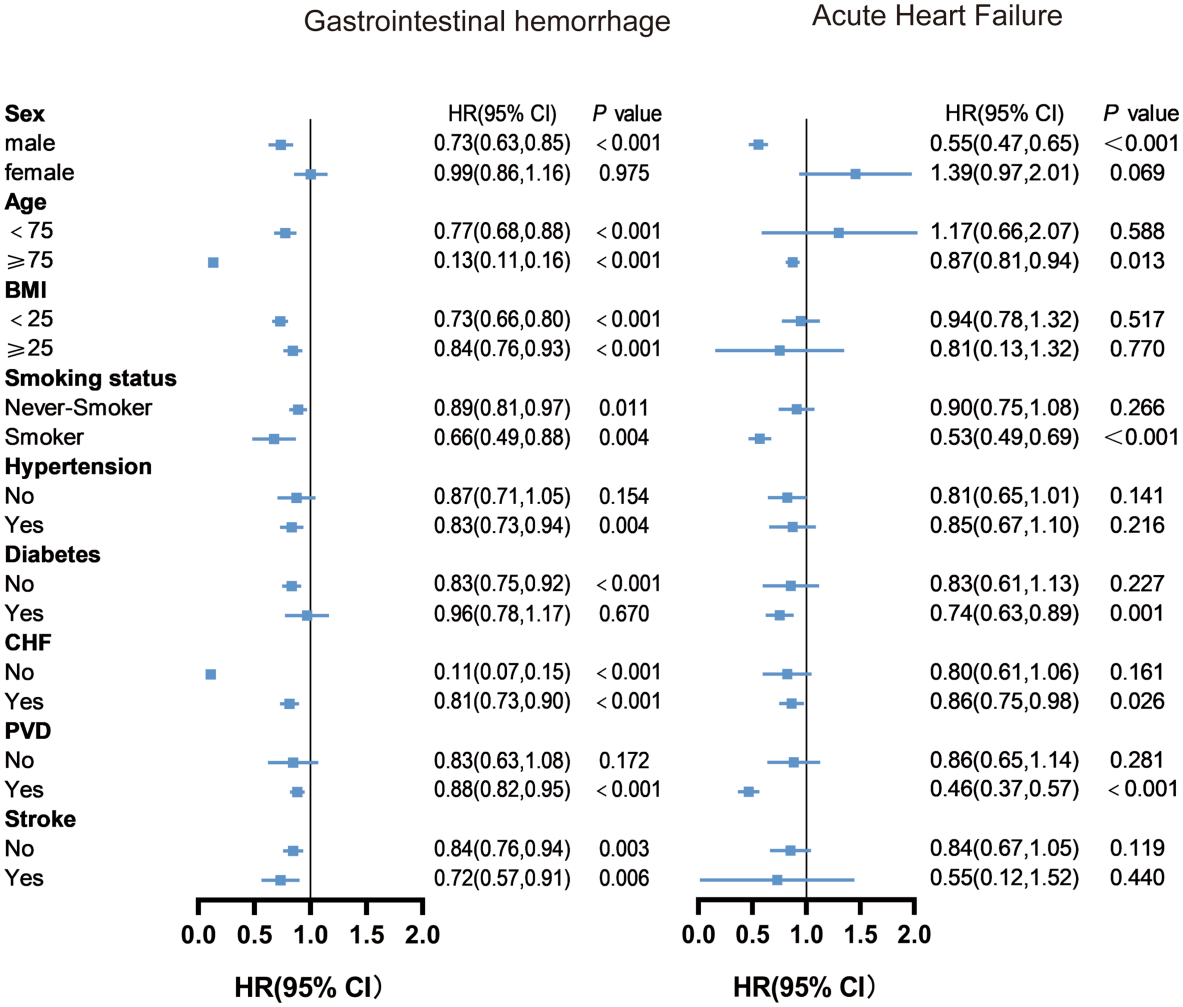
Subgroup analysis results of changes in albumin levels on gastrointestinal hemorrhage and acute heart failure. Model adjusted for all variables except the subgroup variable, including age, sex, BMI, smoking history, hypertension, diabetes, congestive heart failure, peripheral vascular disease, stroke, aspirin, clopidogrel, ticagrelor, statins, anticoagulants, ACEI/ARB, and PCI (For gastrointestinal bleeding, a history of gastrointestinal bleeding was added; For acute heart failure, AMI classification and Killip classification were added). BMI, Body Mass Index; CHF, Congestive Heart Failure; PVD, Peripheral Vascular Disease;ACEI, Angiotensin-Converting Enzyme Inhibitor; ARB, Angiotensin II Receptor Blocker; PCI, Percutaneous Coronary Intervention.

Herein, we conducted stratified analyses to ascertain if the association between albumin changes and outcomes of older AMI patients was consistent across several subgroups (Supplementary Figure 2 and Figures 2, 3). As shown in the figure, albumin changes were associated with all-cause death and cardiac death among participants with male, age ≥ 75, hypertension, non-diabetes, congestive heart failure, non- PVD, and non-stroke. Albumin changes were associated with gastrointestinal hemorrhage among patients with male, age ≥ 75, BMI < 25 or ≥ 25, smoker or not, hypertension, non-diabetes, congestive heart failure or not, PVD, and stroke or not. Albumin changes were associated with acute heart failure among patients with male, age ≥ 75, smoker, diabetes, congestive heart failure, and PVD.

## 4 Discussion

In this observational retrospective study, we tried to examine the optimal albumin changes associated with in-hospital outcomes in older patients with AMI. A S-shaped association between albumin changes and in-hospital mortality was found in the cohort. The correlations between albumin changes and mortality were different according to the inflection of roughly −3.27 g/L and 0.92/L. And albumin changes were negatively associated with gastrointestinal bleeding and acute heart failure. The association was reliable and independent of potential covariates and confounders.

Albumin has long been recognized as an important biomarker of nutritional status and inflammation (12). Previous studies have demonstrated that low albumin levels are associated with poor outcomes in AMI patients (13), yet the optimal threshold for albumin changes has not been fully defined. Our study addresses this gap by using restricted cubic splines to model the relationship between albumin changes and mortality, revealing a non-linear association that suggests both excessive decreases and increases in albumin are linked to worse outcomes. One of the key strengths of this study lies in the identification of specific thresholds for albumin changes that correlate with mortality. Previous research has primarily focused on static albumin levels or simple cutoffs, but our findings indicate that it is not only the absolute albumin concentration, but the magnitude of its change that plays a crucial role in patient outcomes. When albumin decreases by more than 3.27 g/L or increases by more than 0.92 g/L, the changes were negatively associated with AMI mortality. However, when the changes were within a more moderate range, mortality was lower.

The observed associations between albumin changes and gastrointestinal bleeding and acute heart failure further underscore the complexity of albumin’s role in AMI prognosis. Previous studies have shown that low albumin levels are linked to higher risks of bleeding and heart failure in AMI patients, supporting our findings (10, 14). However, our study is the first to show that fluctuations in albumin levels, rather than just low levels, are also significantly correlated with these complications. The relationship between albumin changes and complications such as gastrointestinal bleeding and acute heart failure can be explained by albumin’s role in maintaining oncotic pressure and vascular integrity (15). Significant changes in albumin levels could indicate severe underlying physiological stress, potentially triggering compensatory mechanisms that stabilize vascular integrity and reduce the risk of complications. Conversely, insufficient changes in albumin may fail to activate these protective mechanisms, leaving patients vulnerable to adverse events. In addition to its effects on vascular integrity, albumin also has well-documented anti-inflammatory and antioxidant properties (16, 17). In the context of AMI, substantial changes in albumin levels may reflect an acute phase response characterized by the upregulation of cytokines and antioxidants. This response likely serves to mitigate the damaging effects of inflammation and oxidative stress during the acute phase of myocardial injury. However, if the changes in albumin are insufficient to trigger these mechanisms, persistent oxidative stress and inflammation can contribute to poor outcomes. The failure to activate adequate compensatory responses could explain why small changes in albumin, especially within narrower thresholds, are associated with worse prognosis.

Our subgroup analysis revealed that, for AMI patients aged >75 years, an increase in albumin levels was associated with a stronger protective effect against gastrointestinal bleeding and AHF. This phenomenon can be explained by the unique clinical characteristics of older AMI patients and their higher baseline risk of complications. With increasing age, older patients with acute coronary syndrome often present with atypical symptoms, which pose challenges to timely diagnosis and treatment. Previous studies have shown that 44% of older myocardial infarction patients do not report chest pain as their primary symptom, and approximately 40% of ST-elevation myocardial infarction patients in this age group do not experience chest pain at all (18). These atypical presentations frequently lead to delays in diagnosis and treatment, resulting in poorer outcomes for this population. Moreover, AMI patients aged ≥75 years face significantly higher risks of mortality and heart failure (19, 20), as well as an increased risk of bleeding complications during the acute phase due to multiple comorbidities and the use of anticoagulants (21) Given the high baseline risks in this population, an increase in albumin levels may exert a stronger protective effect through mechanisms such as stabilizing vascular integrity, maintaining oncotic pressure, and reducing inflammation.

Our study also provides important insights into the potential clinical utility of albumin supplementation or careful albumin monitoring in AMI patients, particularly those with significant changes in albumin levels. Given the established link between low albumin levels and adverse outcomes, interventions aimed at stabilizing albumin levels could be beneficial in preventing mortality and complications. Specifically, patients with albumin decreases greater than 3.27 g/L may benefit from targeted supplementation, potentially reducing their risk of poor outcomes.

Although our study provides valuable insights, it also has limitations. The primary limitation is the potential for selection bias due to the strict inclusion and exclusion criteria. A significant number of patients were excluded, particularly those with missing albumin data. This may have resulted in a less representative study population, potentially affecting the generalizability of our findings and underestimating the true association between albumin changes and clinical outcomes. Additionally, while we adjusted for many potential confounders, it is still possible that unmeasured variables, such as the timing of albumin measurements or specific treatment interventions, influenced the results. Future studies with a broader population and more comprehensive data collection are needed to validate our findings.

In conclusion, our study provides novel insights into the dynamic role of albumin changes in the prognosis of older patients with acute myocardial infarction. We found an S-shaped relationship between albumin changes and mortality, with optimal thresholds identified for both decreases and increases in albumin levels. These findings suggest that albumin changes, rather than static albumin levels, may be an important prognostic factor in AMI patients. Clinicians should consider monitoring albumin levels and using appropriate interventions, such as albumin supplementation, particularly in patients with significant fluctuations in albumin.

## 5 Conclusion

The present study found a S-shaped association between the albumin changes and mortality of older patients with AMI, with the inflection of roughly −3.27 g/L and 0.92/L. And changes in albumin levels are negatively correlated with gastrointestinal bleeding and acute heart failure. The potential role of albumin changes in AMI patients should be considered during the treatment.

## Data Availability

The original contributions presented in the study are included in the article/Supplementary material, further inquiries can be directed to the corresponding author.
